# Activation of angiotensin‐converting enzyme 2/angiotensin (1–7)/mas receptor axis triggers autophagy and suppresses microglia proinflammatory polarization via forkhead box class O1 signaling

**DOI:** 10.1111/acel.13480

**Published:** 2021-09-16

**Authors:** Ruili Dang, Mengqi Yang, Changmeng Cui, Changshui Wang, Wenyuan Zhang, Chunmei Geng, Wenxiu Han, Pei Jiang

**Affiliations:** ^1^ Institute of Clinical Pharmacy and Pharmacology Jining First People’s Hospital Jining Medical University Jining China; ^2^ Department of Neurosurgery Affiliated Hospital of Jining Medical University Jining China; ^3^ Department of Pharmacy Zhongshan Affiliated Hospital of Zhongshan University Zhongshan China

**Keywords:** autophagy, FOXO1, neuroinflammation, renin‐angiotensin system

## Abstract

Brain renin‐angiotensin (Ang) system (RAS) is implicated in neuroinflammation, a major characteristic of aging process. Angiotensin (Ang) II, produced by angiotensin‐converting enzyme (ACE), activates immune system via angiotensin type 1 receptor (AT1), whereas Ang(1–7), generated by ACE2, binds with Mas receptor (MasR) to restrain excessive inflammatory response. Therefore, the present study aims to explore the relationship between RAS and neuroinflammation. We found that repeated lipopolysaccharide (LPS) treatment shifted the balance between ACE/Ang II/AT1 and ACE2/Ang(1–7)/MasR axis to the deleterious side and treatment with either MasR agonist, AVE0991 (AVE) or ACE2 activator, diminazene aceturate, exhibited strong neuroprotective actions. Mechanically, activation of ACE2/Ang(1–7)/MasR axis triggered the Forkhead box class O1 (FOXO1)‐autophagy pathway and induced superoxide dismutase (SOD) and catalase (CAT), the FOXO1‐targeted antioxidant enzymes. Meanwhile, knockdown of MasR or FOXO1 in BV2 cells, or using the selective FOXO1 inhibitor, AS1842856, in animals, suppressed FOXO1 translocation and compromised the autophagic process induced by MasR activation. We further used chloroquine (CQ) to block autophagy and showed that suppressing either FOXO1 or autophagy abrogated the anti‐inflammatory action of AVE. Likewise, Ang(1–7) also induced FOXO1 signaling and autophagic flux following LPS treatment in BV2 cells. Cotreatment with AS1842856 or CQ all led to autophagic inhibition and thereby abolished Ang(1–7)‐induced remission on NLRP3 inflammasome activation caused by LPS exposure, shifting the microglial polarization from M1 to M2 phenotype. Collectively, these results firstly illustrated the mechanism of ACE2/Ang(1–7)/MasR axis in neuroinflammation, strongly indicating the involvement of FOXO1‐mediated autophagy in the neuroimmune‐modulating effects triggered by MasR activation.

## Graphical abstract

The neuroimmune‐regulatory mechanism of ACE2/Ang(1–7)/MasR axis. ACE2 cleaves AngII into Ang(1–7), which than binds with MasR. The activation of MasR triggers FOXO1 translocation and induces autophagic process, thereby accelerating the clearance of NLRP3 inflammasome.

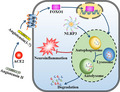

## 1 INTRODUCTION

Sustained neuroinflammation is one of the most striking hallmarks shared by various neurodegenerative disorders, such as Alzheimer's disease (AD) and Parkinson's disease (PD). Excessive inflammatory response would trigger oxidative damage and coordinately lead to neural aging and cell apoptosis (Ransohoff et al., 2015). The predominant type of immune cell in the central nervous system (CNS) is microglia, a type of tissue‐resident macrophage (Shaerzadeh et al., 2018). Similar to macrophage, microglia be categorized into proinflammatory M1 phenotype and immunoregulatory M2 phenotype, which is responsible for the production of proinflammatory or anti‐inflammatory cytokines, respectively (Tang & Le, 2016). Emerging evidence indicates that microglial polarization and generation of reactive oxidative species (ROS) are tightly related to brain intrinsic renin–angiotensin system (RAS) (Mowry & Biancardi, 2019). The major components of RAS now are recognized to be widely distributed throughout the brain and orchestrate a complex impact on the neurological function. Angiotensin‐converting enzyme (ACE) cleaves angiotensin I into angiotensin II (Ang II), which is then converted into Ang(1–7) by ACE2, an isoform of ACE. Ang II and Ang(1–7) are the ligands of angiotensin type 1 receptor (AT1) and Mas receptor (MasR), respectively, exerting opposing biological actions (Alenina & Bader, 2019). Our recent study showed that overactivation of ACE/AngII/AT1 pathway is involved in the inflammatory activation following lipopolysaccharide (LPS) exposure (Gong et al., 2019). However, the excessive inflammatory response can be counterbalanced by the RAS protective component, Ang(1–7), which exerts its effects through MasR, a G protein‐coupled receptor, in various tissues, including the kidneys, cardiovascular system, and brain (Santos et al., 2018). Although it seems very likely the balance between the two arms of RAS plays an essential role in neuroinflammation, there is no research that systematically examined the expression of these RAS members during inflammatory process and the immune‐regulatory mechanisms remain largely obscure.

Autophagy is a conserved evolutionarily homeostatic cellular process, which senses intracellular stress and efficiently mount a response to cope with the damage through sequestration and degradation of damaged organelles and compromised proteins (Kulkarni et al., 2019). Autophagy controls immune cell function at least in part by targeting proinflammatory cytokines and inflammasome structures for degradation (Han et al., 2019). It is of interest to note that RAS and autophagy share converging regulatory effects on neuronal function, including neuroinflammation, oxidative cascade, and synaptic plasticity. Previous research also repeatedly demonstrated the interrelationship between RAS and autophagy in various tissues and cell types (Zhang et al., 2019). Except that, Forkhead box class O1 (FOXO1), the crucial transcriptional factor of autophagic genes, is the downstream of ACE2/Ang(1–7)/MasR axis (Alenina & Bader, 2019; Verano‐Braga et al., 2012). These data allow us to hypothesize that MasR activation may trigger the autophagic process through FOXO1 to influence the neuroinflammatory response.

Based on these clues, the present study firstly evaluated the brain RAS in response to both acute and sustained inflammatory stimuli. Then, we try to explore the immune‐regulatory mechanism of ACE2/Ang(1–7)/MasR axis by analyzing the interaction between ACE2/Ang(1–7)/MasR axis and autophagy in the development and treatment of neuroinflammation.

## 2 RESULTS

### 2.1 Repeated lipopolysaccharide exposure shifts the balance of brain renin–angiotensin system

**FIGURE 1:**
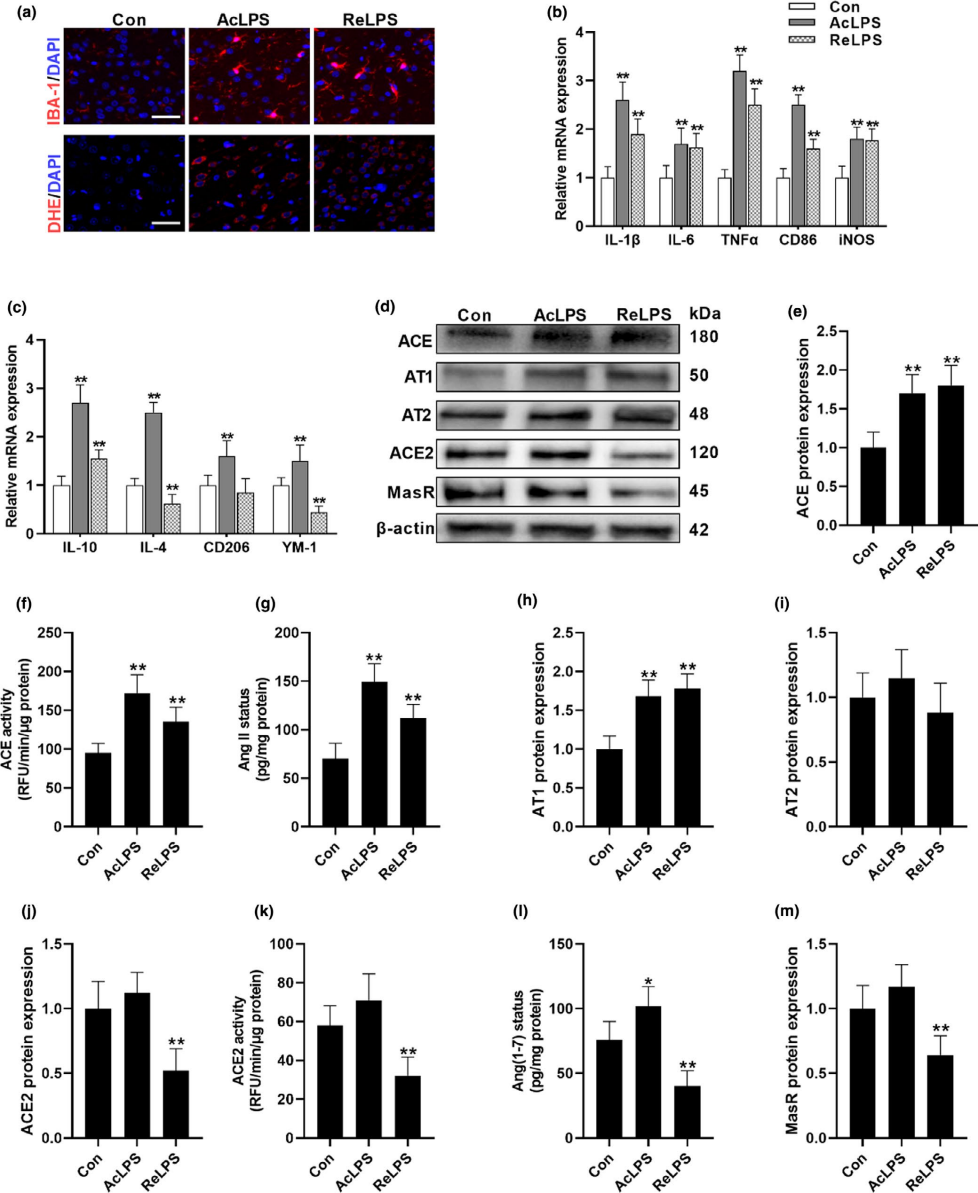
LPS exposure shifts the balance of brain RAS. (a) The impacts of acute LPS (AcLPS) or repeated LPS (ReLPS) exposure on microglial activation (IBA‐1 staining) and ROS generation (DHE staining) in the brain cortex of C57BL/6 mice. (b) Biomarkers of microglial M1 phenotype mRNA expression. (c) Biomarkers of microglial M2 phenotype mRNA expression. (d–m) Alterations of ACE/AngII/AT1 and ACE2/Ang(1–7)/MasR pathways following LPS treatment. (d) Representative western blots. ACE protein expression (e) and activity (f). (g) AngII concentration. (h) AT1 protein expression. (i) AT2 protein expression. ACE2 protein expression (j) and activity (k). (L) Ang(1–7) concentration. (m) MasR protein expression. Scale bar = 50 μm. Data are means ± SD (*n* = 7–9). *p < 0.05, **p < 0.01 compared to control group

As previously reported (Guo et al., 2016; Yang et al., 2018), both acute and repeated LPS treatment led to marked microglial activation (Figure 1a). In accordance, acute treatment group (AcLPS) and repeated LPS (ReLPS) group both exhibited increased expression of M1‐secreted proinflammatory mediators, including interleukin‐1β (IL‐1β), IL‐6, tumor necrosis factor α (TNFα), CD86, and inducible nitric oxide synthase (iNOS) (Figure 1b). Intriguingly, acute LPS treatment seems to cause more significant impacts on the expression of proinflammatory mediators than ReLPS group. These findings suggest that the immune reactivity of microglial cells tends to be lowered which might be attributed to the endotoxin tolerance following repeated LPS exposure. Similarly, a study also showed that in spite of the significant increase of the proinflammatory cytokines in rat hippocampus following acute LPS administration, 3 months of LPS treatment only elevates IL‐1β protein levels but has no effect on IL‐6 and decreases TNF‐α status (François et al., 2014). Notably, one dose of LPS compensatorily induced the biomarkers of M2 phenotype, whereas ReLPS shifted microglia polarization from M2 to M1 phenotype with suppressed M2‐secreted IL‐4, CD206, and YM‐1, except that the IL‐10 expression was still higher compared with the Con group but was much lower than AcLPS group (Figure 1c), suggesting the LPS‐induced compensatory response was exhausted or compromised following sustained LPS stimuli.

Given the critical role of RAS in microglial polarization, we further assessed the brain RAS in response to inflammatory stress (Figure 1d–m). As we previously reported (Gong et al., 2019), both acute and sustained LPS challenge remarkably activate the classical RAS, ACE/AngII/AT1 axis, with increased ACE and AT1 expression and total ACE activity, generating more AngII. Notably, LPS had no significant effect on AT2 expression. Although acute LPS exposure did not alter ACE2 and MasR status, the Ang(1–7) concentration was elevated in AcLPS group, probably due to the increased AngII, which is the substrate for ACE2 to produce Ang(1–7). However, in the conditions of chronic inflammation, the protective arms of RAS and ACE2/Ang(1–7)/MasR axis, were inhibited. Considering the fact that ACE/AngII/AT1 and ACE2/Ang(1–7)/MasR axis may promote microglial M1 proinflammatory polarization and M2 immune‐regulatory polarization, respectively, these data raise the possibility that the balance of brain RAS might be critical to maintaining the homeostasis of brain immune system.

### 2.2 Angiotensin‐converting enzyme 2/angiotensin (1–7)/mas receptor axis activation possesses neuroprotective actions

**FIGURE 2:**
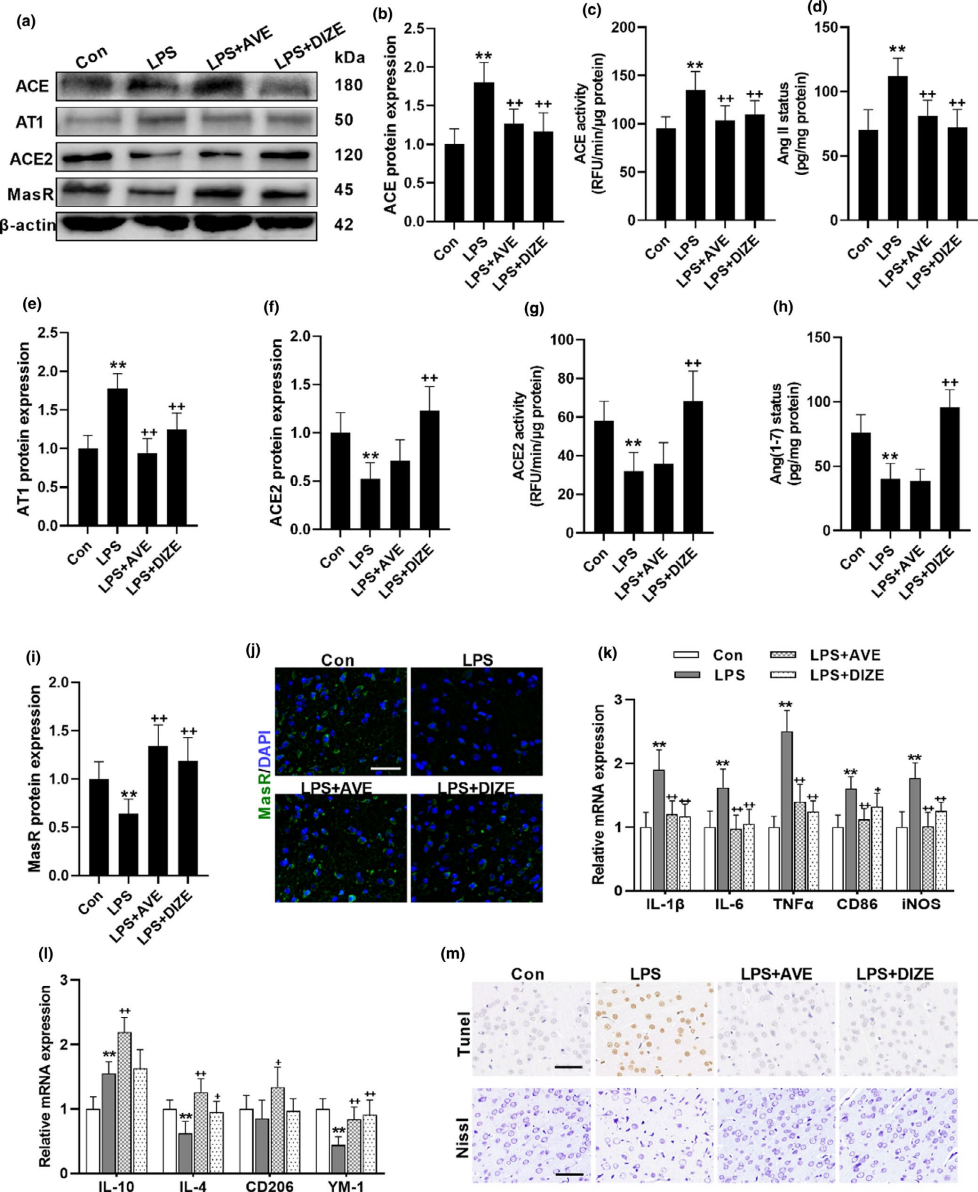
MasR activation restores LPS‐induced overactivation of ACE/AngII/AT1 axis and contains neuroprotective properties. (a–j) The impacts of MasR agonist, AVE, and the ACE2 activator, DIZE, on brain RAS following repeated LPS treatment. (a) Representative western blots. ACE protein expression (b) and activity (c). (d) AngII concentration. (e) AT1 protein expression. ACE2 protein expression (f) and activity (g). (h) Ang(1–7) concentration. MasR protein expression (i) and immunofluorescence staining (j). (m) Representative images of TUNEL and Nissl staining. (k, l) mRNA expression of biomarkers of microglial M1 phenotype (k) and M2 phenotype (l). Scale bar = 50 μm. Data are means ± SD (*n* = 7). **p < 0.01 compared to control group. ^+^ *p* < 0.05, ^++^ *p* < 0.01 compared to LPS group

To restore the balance of brain RAS, the selective MasR agonist, AVE, and the ACE2 activator, diminazene aceturate (DIZE), were used to potentiate the ACE2/Ang(1–7)/MasR axis. Our data showed that MasR activation either by AVE or by DIZE treatment mitigated LPS‐induced ACE/AngII/AT1 overactivation, restoring the balance of brain RAS from ACE/AngII/AT1 side to the ACE2/Ang(1–7)/MasR side in the context of neuroinflammation (Figure 2a–j). DIZE effectively induced the expression, and activity of ACE2 facilitating the biotransformation from AngII to Ang(1–7). Although AVE had no effect on ACE2 and Ang(1–7) status, as a selective MasR agonist, both AVE and DIZE mitigated LPS‐induced decline of MasR expression. Meanwhile, AVE or DIZE ameliorated the increased expression of M1 biomarkers (Figure 2k) and the decrease of M2 biomarkers (Figure 2l) caused by LPS stimuli, triggering the microglial polarization from M1 phenotype to M2 phenotype. Additionally, we further evaluated neuroprotective effects of ACE2/Ang(1–7)/MasR activation by using the TUNEL method and the Nissl staining (Figure 2m). Sustained LPS exposure induced the abundance of TUNEL‐positive cells in the brain cortex, which was restored by AVE or DIZE administration. Likewise, the neural cells were pronouncedly shrunken, irregularly arranged, and weakly stained in LPS group, indicating the loss of Nissl bodies, whereas cotreatment with AVE or DIZE increased the number of viable neurons with deeply stained and regularly arranged forms, which indicates potential strong neuroprotective and immune‐regulatory properties of ACE2/Ang(1–7)/MasR axis.

### 2.3 Mas receptor activation promotes Forkhead box class O1‐autophagy pathway

**FIGURE 3:**
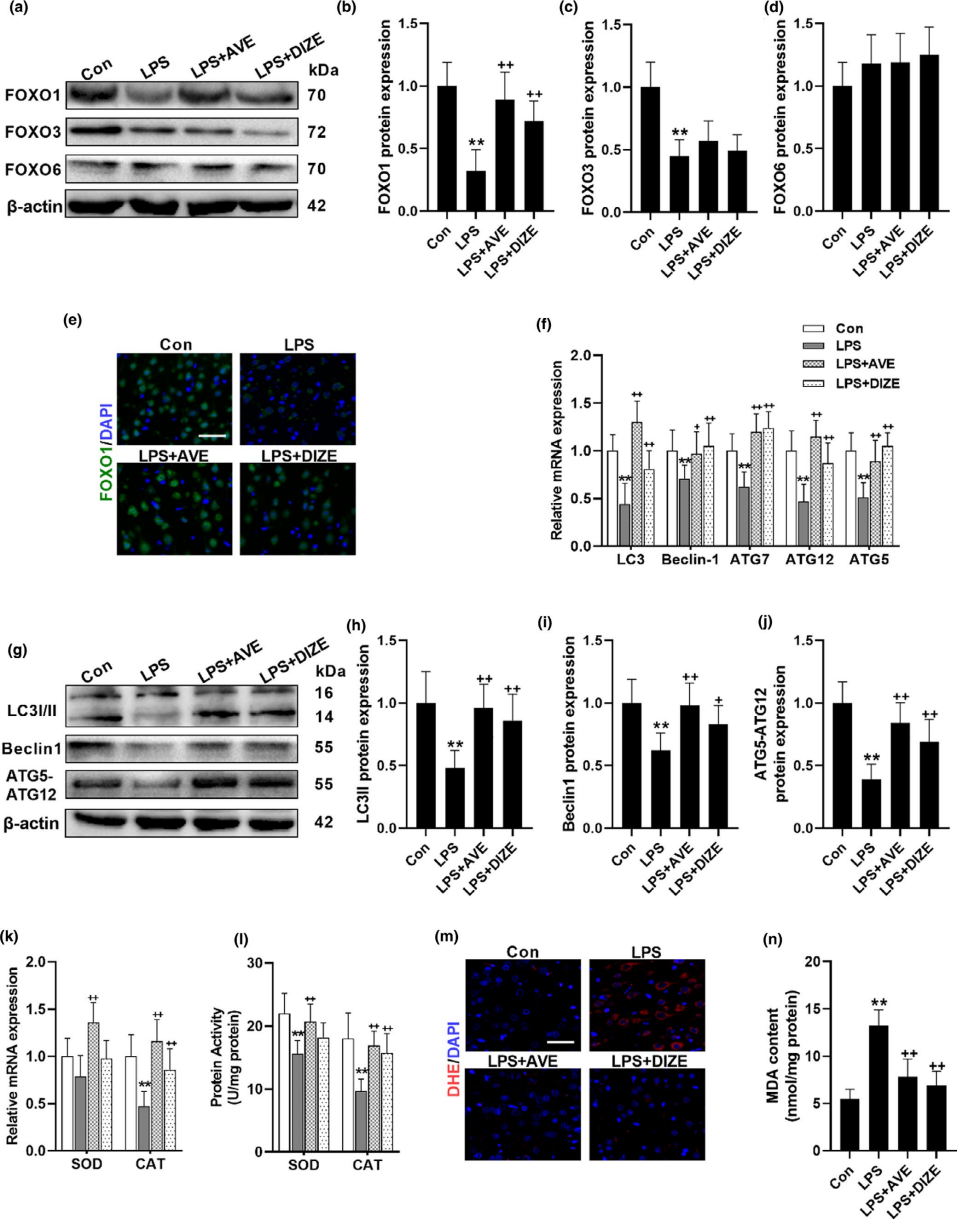
MasR activation triggers FOXO1 signaling and promotes autophagy. (a–e) The impacts of MasR agonist, AVE, and the ACE2 activator, DIZE, on the expression of FOXOs following repeated LPS treatment. (a) Representative western blots of FOXOs. Statistical graphs of protein expression of FOXO1 (b), FOXO3 (c), and FOXO6 (d). (e) Representative images of immunofluorescence assays of FOXO1. (f–j) The impacts of MasR agonist, AVE, and the ACE2 activator, DIZE, on autophagic genes. (f) mRNA expression of autophagic genes. (g) Representative western blots of autophagic genes. Statistical graphs of protein expression of LC3II (h), Beclin1 (i), and ATG5‐ATG12 (j). mRNA expression (k) and protein activity (l) of FOXO downstream antioxidant enzymes. (m) Representative images of DHE immunofluorescence assays. (n) MDA concentration. Scale bar = 50 μm. Data are means ± SD (*n* = 7). **p < 0.01 compared to control group. ^++^ *p* < 0.01 compared to LPS group

To further explore the underlying mechanism, the impacts of MasR activation on FOXO‐autophagic signaling were assessed. Our data showed that LPS inhibited both FOXO1 and FOXO3 expression, whereas AVE or DIZE only relieved the LPS‐induced decrease of FOXO1 expression but had no effect on the LPS‐induced decrease of FOXO3 status (Figure 3a–c). Meanwhile, both LPS and the treatment of AVE or DIZE did not affect FOXO6 expression (Figure 3d). In support, AVE and DIZE treatment also induced the abundance and nuclear translocation of FOXO1 (Figure 3e), which is in line with previous reports that FOXO1 is a major downstream target of ACE2/Ang(1–7)/MasR axis (Verano‐Braga et al., 2012). FOXOs can directly bind to the promoters of autophagic genes and control autophagosomes formation and their fusion with lysosomes (Zhang et al., 2015). As expected, while LPS compromised FOXO1‐autophagic signaling, AVE and DIZE treatment induced both transcriptional and translational status of autophagic genes, such as LC3II, Beclin‐1, and ATG5‐ATG12 (Figure 3f–j). Except that, the mRNA expression and the total activity of the antioxidant enzymes, SOD and CAT, which also are targeted by FOXO1, were enhanced by AVE compared with LPS‐treated group (Figure 3k,l). Likewise, DIZE also alleviated the decreased mRNA expression and protein activity of CAT caused by repeated LPS exposure. The autophagy and the antioxidant enzymes constitute a major ROS‐scavenging system and thereby the activation of FOXO1 markedly attenuated LPS‐induced ROS accumulation (Figure 3m). In support, the elevated status of lipid peroxidation products, malondialdehyde (MDA), caused by LPS treatment was also restored by both AVE and DIZE treatment (Figure 3n).

**FIGURE 4:**
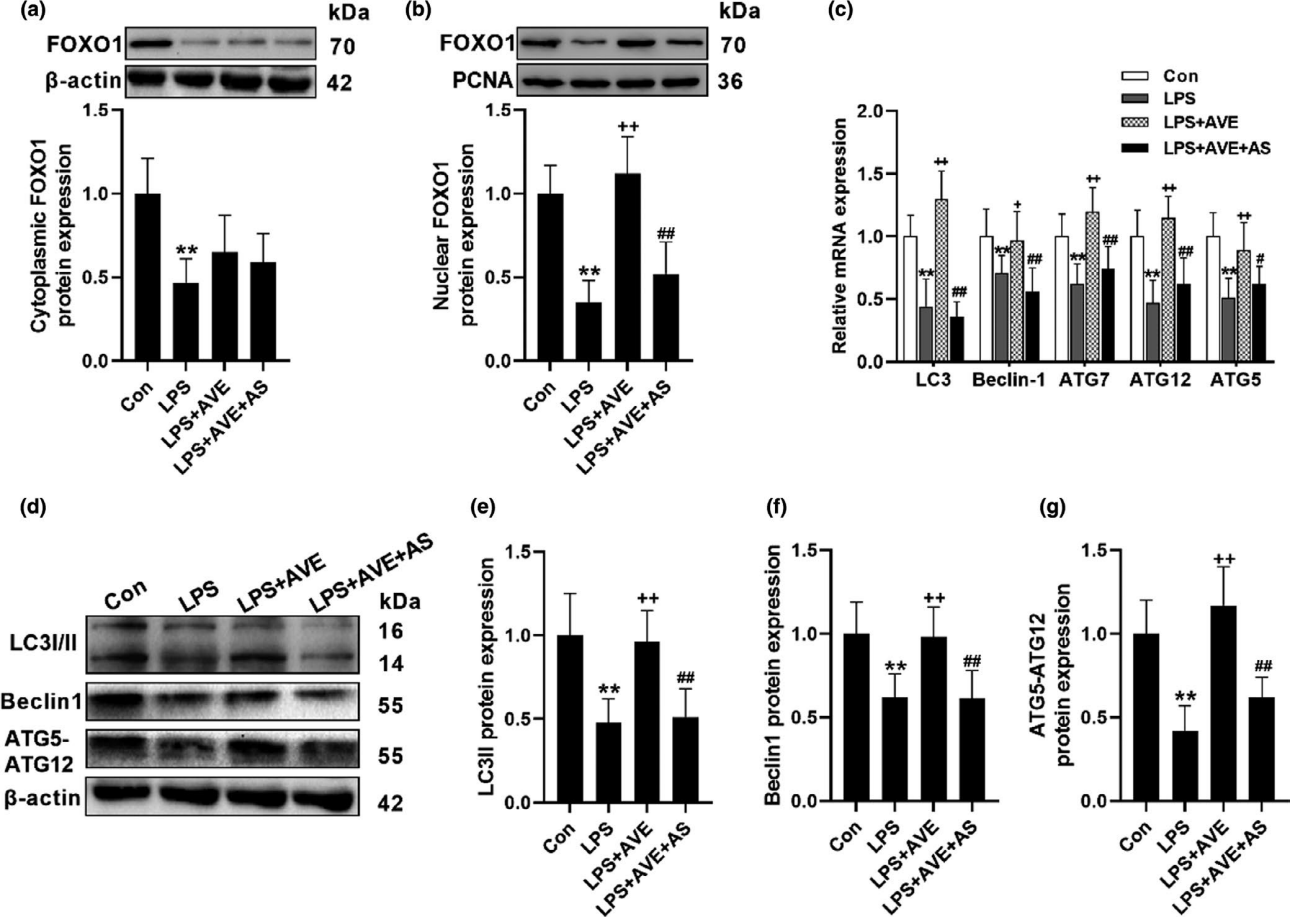
MasR activation promotes autophagy via FOXO1 signaling in mice brain cortex. (a, b) The selective FOXO1 inhibitor, AS, suppressed AVE‐induced FOXO1 translocation. (c) AS abrogated AVE‐induced transcriptional status of autophagic genes. (d–g) Representative western blots (d) and statistical graphs of protein expression of LC3II (e), Beclin (f), and ATG5‐ATG12 (g). Data are means ± SD (*n* = 7). **p < 0.01 compared to control group. ^++^ *p* < 0.01 compared to LPS group. ^#^ *p* < 0.05, ^##^ *p* < 0.01 compared to LPS + AVE group

**FIGURE 5:**
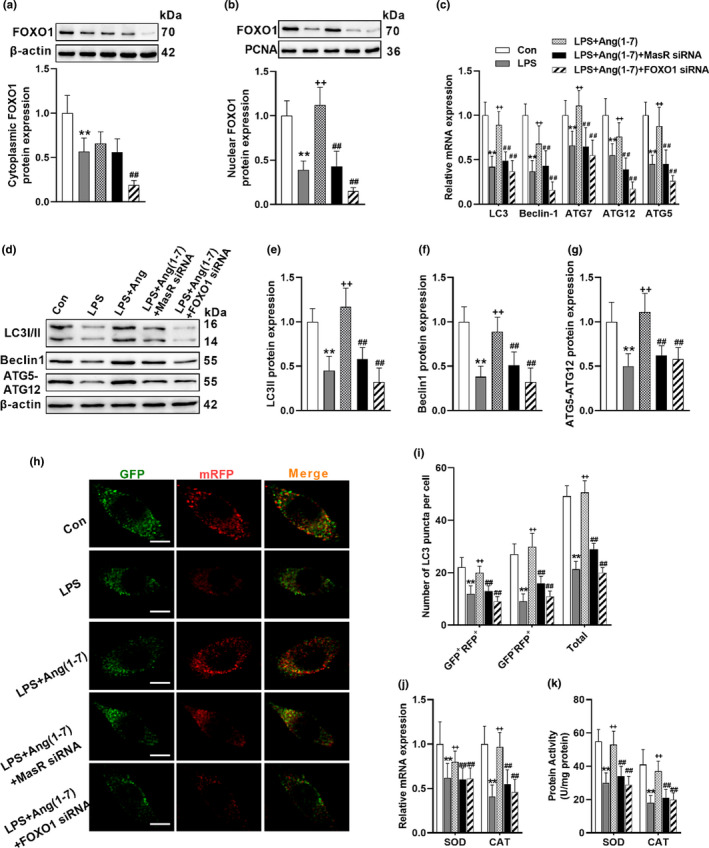
Ang(1–7) promotes autophagy via FOXO1 signaling in BV2 cells. (a, b) Knockdown either MasR or FOXO1 by using siRNAs suppressed Ang(1–7)‐induced FOXO1 translocation. (c) MasR siRNA and FOXO1 siRNA both abrogated Ang(1–7)‐induced transcriptional status of autophagic genes. (d–g) Representative western blots (d) and statistical graphs of protein expression of LC3II (e), Beclin (f), and ATG5‐ATG12 (g). (h) Immunofluorescence staining of microglial cells transfected with mRFP‐GFP‐LC3 adenovirus. (i) Calculated numbers of autophagosome (GFP^+^RFP^+^ yellow puncta) and autolysosome (GFP^−^RFP^+^ red puncta) numbers. (j, k) mRNA expression (j) and protein activity (k) of FOXO downstream antioxidant enzymes. Scale bar = 5 μm. Data are means ± SD (*n *= 6). *p < 0.05, **p < 0.01 compared to control group. ^+^ *p* < 0.05, ^++^ *p* < 0.01 compared to LPS group. ^#^ *p* < 0.05, ^##^ *p* < 0.01 compared to LPS+Ang(1–7) group

To confirm the mechanism, the selective FOXO1 inhibitor AS1842856 (AS), was coadministered with AVE in the animals. AS blocked AVE‐induced translocation of FOXO1 into the nucleus (Figure 4a,b), thereby effectively compromised AVE‐induced mRNA and protein expression of autophagic genes in the conditions of chronic inflammation (Figure 4c–g). Considering microglia are the brain‐resident macrophages which mediate the neuroinflammatory response, we further used BV2 murine microglial cells to explore the mechanism. SiRNAs were used to selectively knock down MasR and FOXO1. After 48 h transfection, both protein and mRNA expression of the related target genes were decreased by the MasR siRNA and FOXO1 siRNA (Figure S1a–e). In accordance, suppressing MasR in BV2 cells inhibited Ang(1–7)‐induced FOXO1 translocation and activation. Similarly, FOXO1 siRNA pretreatment also suppressed FOXO1 expression in both cytoplasm and nucleus. In line with the in vivo results, interfering with MasR or FOXO1 using the siRNAs abrogated the Ang(1–7)‐induced the expression of autophagic genes (Figure 5c–g). We then move forward to investigate the autophagic flux using mRFP‐GFP‐LC3 adenovirus. The fusion of autophagosomes with acidic lysosomes reduces the pH‐sensitive fluorescence of GFP, whereas the RFP fluorescence signal is stable under acidic conditions (Li et al., 2016). Therefore, autophagosomes were observable as yellow puncta (GFP^+^RFP^+^) and autolysosomes appeared as red puncta (GFP^−^RFP^+^). The abundance of yellow puncta and red puncta was synchronously decreased by LPS exposure, whereas Ang(1–7) pretreatment induced both autophagosomes and autolysosomes formation (Figure 5h,i). However, MasR siRNA and FOXO1 siRNA administration compromised the stimulating effects of Ang(1–7) on autophagic flux and antioxidant enzymes (Figure 5j,k). In combination with the Western and PCR data, these results all led to the conclusion that MasR activation triggers autophagy through FOXO1 signaling.

### 2.4 The anti‐inflammatory effects of angiotensin‐converting enzyme 2/angiotensin (1–7)/mas receptor axis is mediated by Forkhead box class O1‐autophagy pathway signaling

**FIGURE 6:**
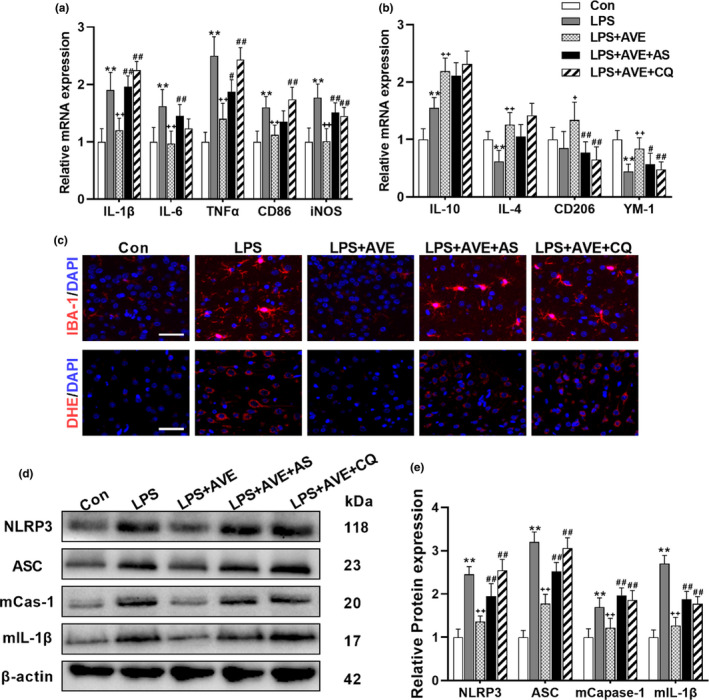
Inhibition of FOXO1 or autophagy compromised the antioxidative and anti‐inflammatory actions of AVE in mice. (a, b) The selective FOXO1 inhibitor, AS, and the autophagy inhibitor, CQ, abrogated the immune‐regulatory effect of AVE on microglial polarization. (c) AS and CQ both inhibited AVE‐induced alleviation of microglial activation (IBA‐1 staining) and ROS generation (DHE staining) following LPS exposure. (d, e) Representative western blots (d) and statistical graphs (e) of the major components of NLRP3 inflammasomes. Scale bar = 50 μm. Data are means ± SD (*n *= 6–7). *p < 0.05, **p < 0.01 compared to control group. ^+^ *p* < 0.05, ^++^ *p* < 0.01 compared to LPS group. ^#^ *p* < 0.05, ^##^ *p* < 0.01 compared to LPS + AVE group

Considering the pleiotropic actions of FOXO1 and autophagy signaling, we next tried to prove their involvement in the neurological activities of ACE2/Ang(1–7)/MasR axis. Our data showed that cotreatment with AS or CQ revoked the immune‐regulatory effects of AVE on microglial polarization (Figure 6a,b) and activation (Figure 6c). Additionally, both ROS and NLRP3 inflammasome components are targeted by autophagy (Su et al., 2019). While MasR activation accelerated the clearance of LPS‐induced ROS (Figure 6c) and NLRP3 inflammasome (Figure 6d,e), inhibition of FOXO1‐autopahgy pathway reversed the antioxidant effects of AVE and induced the accumulation of NLRP3 inflammasome, resulting in aggravated cleavage of IL‐1β, which may further reinforce the proinflammatory activation. Similar results were also observed in BV2 cells. Ang(1–7) facilitated the autophagic degradation of ASC, the major component of NLRP3 inflammasome, reducing the ASC distribution but increasing LC3 distribution compared with LPS‐treated group (Figure S2a). These effects were totally blocked by AS, but CQ increased the colocalization of LC3 and ASC fluorescence indicating that while AS suppressed autophagosomes formation, CQ effectively induced autophagosomes accumulation and consequently suppressed ASC degradation (Figure S2a). As expected, inhibition of FOXO1‐autopahgy abrogated the anti‐inflammatory effect of Ang(1–7), compromising Ang(1–7)‐induced alleviation of LPS‐induced NLRP3 activation (Figure S2b,c) and the shift of microglial polarization from M1 phenotype to M2 phenotype (Figure S2d,e). Together, these data cooperatively suggest that ACE2/Ang(1–7)/MasR axis preserves neuroimmune homeostasis through FOXO1‐autophagy signaling.

## 3 DISCUSSION

Neuroinflammation is a major neuropathological hallmark of neurodegenerative disorders, whereas brain RAS now is found to play an essential role in an inflammatory response and becomes an emerging therapeutic target in brain aging. We previously demonstrated that blocking AT1 receptor by candesartan is effective to attenuate LPS‐induced exaggerated neuroinflammation and AngII is now treated as part of the proinflammatory mediator in microglial response (Gong et al., 2019). Recent studies showed ACE2 activation ameliorates cognitive deficits and protects against neuropathology in the mouse model of Alzheimer's disease (Evans et al., 2020). Likewise, it also has been reported that directly activating MasR by AVE attenuates aging‐related brain inflammation (Jiang et al., 2018). Although this evidence points out that the protective arm of RAS, ACE2/Ang(1–7)/MasR axis, might be a novel target in neuroinflammation and neurodegenerative disease, its potential immune‐regulatory mechanism remains obscure.

The present study firstly examined the impacts of acute and repeated LPS stimuli on brain RAS. The classical ACE/AngII/AT1 was activated in response to both acute and sustained LPS treatment. These results are in line with previous researches showing that AngII is an important mediator of inflammatory process (Gong et al., 2019). Treatment with AngII alone is effective to trigger inflammatory response and oxidative damage in various tissues and cell types and interrupt with ACE/AngII/AT1 axis by ACE inhibitors or AT1 blockers are protective (Abdul‐Muneer et al., 2018; Zhang et al., 2012). By evaluating ACE2/Ang(1–7)/MasR axis, we found that acute LPS treatment had no significant effect on ACE2 and MasR except that Ang(1–7) status was increased which might be attributed to the elevated concentration of AngII, the ACE2 substrate to biotransform into Ang(1–7). On the other hand, prolonged inflammatory condition pronouncedly suppressed ACE2/Ang(1–7)/MasR axis, reducing the protein expression and activity of ACE2 and consequently decreasing the Ang(1–7) status and MasR expression. These data are interesting given most of the neuropathological disorders are associated with chronic inflammatory condition. The shifted balance between ACE/AngII/AT1 and ACE2/Ang(1–7)/MasR axis to the deleterious side induced by the persistently inflammatory milieu may further enhance and sustain the inflammatory and oxidative stress. This may partly explain our finding that the expression of cytokines or mediators of M2 immune‐regulatory phenotype was compensatorily increased following acute LPS exposure, whereas continuous LPS exposure promoted the microglial polarization from M2 to M1 phenotype.

To restore the balance of RAS, the selective MasR agonist, AVE, and the ACE2 activator, DIZE, were used to enhance ACE2/Ang(1–7)/MasR pathway. We found that stimulation of ACE2 and MasR both toned down the LPS‐induced overreaction of ACE/AngII/AT1 pathway. The reciprocal inhibitory effect between the two axes of RAS has been reported in various tissues (Santos et al., 2018). We previously showed the suppressed ACE2/Ang(1–7)/MasR axis in spontaneously hypertensive rats, which is characterized with overactivated ACE/AngII/AT1 pathway in the brain and periphery (Cui et al., 2019). The shift balance of RAS to the deleterious side was also found in the brain of animals with streptozocin‐induced diabetes and Alzheimer's disease (Chen et al., 2017). In support of our findings, a recent study also showed LPS triggers ACE/AngII/AT1 but suppresses ACE2/Ang(1–7)/MasR in retinal pigment epithelium, whereas DIZE reduces the LPS‐induced proinflammatory response through MasR (Tao et al., 2016). Likewise, AVE demonstrated strong antioxidative and neuroprotective actions in subarachnoid hemorrhage (Mo et al., 2019). The present study also shows the neuroprotective effects of MasR activation against LPS‐induced neural loss and apoptosis, which is associated with its immune‐regulatory actions. In line with our findings, activating ACE2 or MasR can mitigate aging‐related neuroinflammation and cognitive decline (Ho & Nation, 2018; Jiang et al., 2018). Stimulation of ACE2/Ang(1–7)/MasR axis by DIZE also ameliorates the behavioral deficits and protects the brain against amyloid pathology, inflammatory overactivation, and oxidative damage in animal models of Alzheimer's disease (Evans et al., 2020; Kamel et al., 2018). Additionally, direct intrastriatal administration of Ang(1–7) can reduce brain lesions and motor deficits in 6‐hydroxydopamine‐induced hemiparkinsonian rats (Rabie et al., 2020). These results all lend weight to the potential beneficial effects of ACE2/Ang(1–7)/MasR axis in the neurodegenerative disorders that are repeatedly demonstrated to be characterized by sustained inflammation and excessive oxidative damage.

We then further explored the underlying mechanism by examining FOXO‐autophagy pathway. In adult mammal brain, the FOXO family members mainly include FOXO1, FOXO3, and FOXO6 (Santo & Paik, 2018). Current evidence has revealed that FOXOs transactivate genes that involve in neurogenesis, oxidative stress, and autophagy, which is critical for the maintenance of brain homeostasis (McLaughlin & Broihier, 2018). Genetic knockout of FOXOs results in age‐dependent neurodegeneration (Murtaza et al., 2017). Intriguingly, recent research also found that FOXO‐deficiency leads to aberrant dendritic morphology and severely impaired autophagic flux, whereas induction of autophagy rescues the abnormities of FOXO‐ablated neurons (Kannangara & Lagace, 2018). In the present study, we found that LPS suppressed FOXO1 and FOXO3, and weakened autophagic flux. These data are in accordance with the recent reports that FOXO1 and FOXO3 are involved in the LPS‐induced impairment of autophagy in the microglia and brain tissues (Lee et al., 2019). It has been found that Ang(1–7) directly activates FOXO1 signaling via MasR, promoting FOXO1 translocation and transcriptional activating downstream genes (11). To our interest, previous research also demonstrated that Ang(1–7) facilitates autophagy in cancer cells and lung fibroblasts (Lin et al., 2018; Pan et al., 2018). These results are in line with our data showing that MasR activation promoted transcriptional and translational levels of autophagic biomarkers. To confirm the role of FOXO1, we further blocked FOXO1 signaling by using the selective inhibitor, AS. We found that AS inhibited FOXO1 translocation and abrogated AVE‐induced autophagic genes. Similarly, by using Ang(1–7) in vitro, we also found that while Ang(1–7) ameliorated the LPS‐induced impairment of FOXO1‐autophagy pathway, suppressing FOXO1 signaling totally blocked Ang(1–7)‐induced autophagy, illustrating a critical mechanism by which Ang(1–7) activates autophagic flux.

We then investigated whether FOXO1‐autophagy signaling is involved in the protective effects of ACE2/Ang(1–7)/MasR pathway, given the importance of autophagy in restraining excessive inflammation and oxidative stress. AS and CQ were used to inhibit FOXO1 and autophagic flux, respectively. As expected, inhibiting FOXO1‐autophagy compromised the anti‐inflammatory and antioxidative effects of MasR activation following LPS challenge. NLRP3 inflammasome, which is indispensable for IL‐1β maturation, is a dominant driver of neuroinflammation (Zhang et al., 2019). Now, it has been found that NLRP3 and IL‐1β itself both are targeted by autophagy (Harris et al., 2017). Therefore, the accelerated autophagic flux driven by MasR signaling may enhance the degradation of these proinflammatory mediators, rebalancing the brain to homeostasis. Moreover, ROS also can trigger NLRP3 inflammasome activation, resulting in sustained and excessive neuroimmune activation (Minutoli et al., 2016). Since autophagy limits inflammasome assembly by eliminating various endogenous stimuli, including dysfunctional mitochondria and ROS, the Ang(1–7)‐induced autophagy may cut off the feed‐forward mechanism between neuroinflammation and oxidative stress by promoting the autophagic clearance of both ROS and NLRP3 inflammasome.

Collectively, the present study evaluated the response of brain RAS to inflammatory stimuli, showing that sustained LPS exposure shifted the balance between ACE/Ang II/AT1 and ACE2/Ang(1–7)/MasR axis to the deleterious side. On the other hand, potentiating ACE2/Ang(1–7)/MasR axis by using either ACE2 activator or MasR agonist promoted autophagic process through FOXO1 and enhanced the clearance of NLRP3 inflammasome and ROS, thereby protecting the brain from LPS‐induced excessive inflammation and oxidative stress. Our data showed the interaction between brain ACE2/Ang(1–7)/MasR axis and autophagy, providing a potential mechanism and raising ACE2 as a novel drug target to restrain uncontrolled neuroimmune overactivation and oxidative stress that repeatedly found in neurodegenerative disorders.

## 4 MATERIALS AND METHODS

### 4.1 Animals

Adult male 8‐week‐old C57BL/6 mice were housed under standard conditions of temperature (23 ± 2°C) and light (12:12 h light/dark cycle), with free access to rodent chow and water. All animal use procedures were carried out in accordance with the Regulations of Experimental Animal Administration issued by the State Committee of Science and Technology of the People's Republic of China, with the approval of the Animal Ethics Committee of our University.

### 4.2 Drug administration

The animals received LPS (Escherichia coli serotype 0111:B4, Sigma‐Aldrich) via intraperitoneal injection at a dose of 500 μg/kg. For AcLPS, the animals were sacrificed 6 h after the injection. To mimic the clinical prolonged impacts of inflammation, ReLPS treatment group was injected every 2 days for a total of seven injections. The dose and treatment duration of LPS was chosen to effectively provoke neuroinflammation without causing immunotolerance based on our previous research (Dang et al., 2018). 3 mg/kg of AVE0991 (Maycogene) or 15 mg/kg of DIZE (Maclin) was given continuously via intraperitoneal injection for 2 weeks to activate ACE2/Ang(1–7)/MasR axis as previously reported (Evans et al., 2020; Mo et al., 2019). To block FOXO1 signaling or autophagy, the selective FOXO1 inhibitor, AS1842856 (Maclin) or the lysosomal inhibitor, chloroquine (Maclin), was administrated daily at the dose of 5 and 30 mg/kg, respectively, for the 2‐week treatment regime (Jiang et al., 2017; Spurthi et al., 2018).

### 4.3 Cell culture and treatment

Murine microglial cell line BV2 was cultured at fully humidified atmosphere of 5% CO_2_ in Dulbecco's modified Eagle's medium (DMEM)/F‐12 with 10% fetal bovine serum (FBS) at 37°C. Before the experiments, BV2 cells were seeded into 24‐well and six‐well plates at a density of 3 × 10^5^ and 1 × 10^6^ cells per well, respectively. Following one day incubation, serum‐free medium (SFM) was added and incubated in SFM for an additional 4 h. For drug treatment, cells were pretreated for 1 h with 100 nM Ang(1–7) before being stimulated with 1 μg/ml LPS for 24 h. AS (1 μM) and CQ (40 μM) were added simultaneously with LPS to block FOXO1 and autophagic process, respectively.

### 4.4 Real‐time PCR

Total RNA was extracted using Trizol reagent (Invitrogen). Quantitative PCR was performed on Bio‐Rad C×96 Detection System (Bio‐Rad) using SYBR green PCR kit (Applied Biosystems) and gene‐specific primers (Table S1). Relative quantitation for PCR product was normalized to β‐actin as an internal standard.

### 4.5 Western blot

For western blotting analysis, total or nuclear protein was prepared and the concentrations were analyzed using the Bradford method. Samples were loaded on precast 12% SDS‐PAGE gels with approximately 50 µg protein in each lane. The following antibodies and concentrations were used over night at 4°C; ACE (Abcam, ab254222, 1:1000), AT1 (Proteintech, 25343‐1‐AP, 1:1000), AT2 (Proteintech, 13436‐1‐AP, 1:1000), ACE2 (Proteintech, 21115‐1‐AP, 1:1000), MasR (Novus, NBP1‐78444, 1:1000), FOXO1 (Proteintech, 18592‐1‐AP; 1:1000), FOXO3 (Proteintech, 10849‐1‐AP; 1:1000), FOXO6 (Proteintech, 19122‐1‐AP; 1:1000), LC3 (Cell signaling, 4108; 1:1000), ATG5‐ATG12 (Cell signaling, 4180; 1:1000), Beclin‐1 (Abcam, ab62557; 1:1000), NLRP3 (Abcam, ab214185; 1:200), ASC (Novus, NBP1‐78977, 1:1000), caspase‐1 (Abcam, ab1872; 1:1000), IL‐1β (Abcam, ab9722; 1:1000), and β‐actin (Proteintech, 66009‐1‐Ig; 1:4000). Following incubation with the HRP‐conjugated secondary antibody, the signals were detected with an ECL kit (Thermo Fisher) and then quantified using Image J software.

### 4.6 Histopathological staining

After sacrifice, the brain tissues were fixed in 10% phosphate‐buffered paraformaldehyde and then embedded in paraffin, prepared for histopathological examination and immunohistochemical staining. For Nissl staining, brain paraffin tissues were sectioned with 4 μm thickness, which were immersed in cresyl violet (0.1%), rinsed in distilled water and differentiated in 95% ethyl alcohol. The presence of apoptosis was assessed by the terminal deoxynucleotidyl transferase‐mediated FITC‐dUTP nick end labeling (TUNEL) method, which detects fragmentation of DNA in the nucleus during apoptotic cell death in situ (Wang et al., 2019). The apoptosis detection assay was performed using a commercially available kit following the manufacturer's protocol (Keygen Biotech).

### 4.7 Angiotensin‐converting enzyme and angiotensin‐converting enzyme 2 activity analysis

The activities of ACE and ACE2 were quantified by their ability to convert their fluorogenic substrates to fluorescent products as reported previously (Wang et al., 2016). In the ACE activity assay, ACE‐specific fluorogenic peptide Abz‐FRK(Dnp)‐P (Enzo Life Sciences) was mixed with the sample in the presence and absence of captopril. Similarly, an ACE2 substrate peptide Mca‐APK(Dnp) (Enzo Life Sciences) in the presence and absence of MLN4760 was used to examine ACE2 activity. Specific enzyme activity was calculated by subtracting the relative fluorescence units (RFU)/min in the presence of the specific inhibitors from total fluorescence without inhibition.

### 4.8 Biochemical parameter assay

The brain cortex was homogenized and centrifuged at 8600 g for 15 min at 4°C. The supernatants were used for the measurement of Ang II and Ang (1–7) by using the commercially available Enzyme Linked Immunosorbent Assay (ELISA) kits (Cusabio). Malondialdehyde (MDA) levels were measured using the thiobarbituric acid reactive substances (TBARS) method. The activities of superoxide dismutase (SOD) and catalase (CAT) were determined using the SOD and CAT assay kits, respectively (Nanjing Jiancheng Bioengineering Institute) according to the manufacture's instructions.

### 4.9 RNA interference

The small interfering RNAs (siRNAs) targeting FOXO1 (sc‐35383), MasR (sc‐62601), and siRNA negative control (sc‐37007) were purchased from Santa Cruz Biotechnology. Bv2 cells were transfected with FOXO1 siRNA, MAS 1 siRNA, and siRNA NC using Lipofectamine 3000 reagent (Invitrogen, L3000015) in Opti‐MEM according to the manufacturer's instructions. After 48 h transection, cells were harvested for RT‐qPCR and western blot to evaluate the efficiency of the siRNA interference.

### 4.10 Cell transfection

BV2 cells were transfected with a tandem fluorescent mRFP‐GFP‐MAP1LC3B adenovirus (Hanbio Biotechnology Co., Ltd) at a MOI of 30 viral particles per cell to observe autophagosome and autolysosome according to the manufacturer's instructions. Six fields were chosen from three different cell preparations. GFP‐ and mRFP‐expressing spots, which were indicated by fluorescent puncta and DAPI‐stained nuclei were counted manually. The number of spots per cell was determined by dividing the total number of spots by the number of nuclei in each field.

### 4.11 Immunofluorescence

For the immunofluorescent study, paraffin‐embedded cortical sections of 4μm thickness were dewaxed in xylol, rehydrated and rinsed in PBS. Antigen retrieval process was carried out by boiling the sections in a citric acid buffer (0.01 mol/L, pH 6.0), followed by incubation with blocking 5% goat serum at 25°C. For immunocytochemistry, cells grown on coverslips were fixed with 4% paraformaldehyde for 10 min. Cells were blocked using 1% BSA + 0.3% (v/v) Triton X‐100 + 0.3 M glycine (1% BSA) at room temperature. The sections or fixed cells were then incubated with the following primary antibodies: anti‐MasR (Novus, NBP1‐78444, 1:30), anti‐FOXO1 (Proteintech, 18592‐1‐AP; 1:100), anti‐IBA‐1 (Abcam, ab178847, 1:200), anti‐LC3 (Cell signaling, 4108; 1:200), or anti‐ASC (Novus, NBP1‐78977, 1:50). Generation of reactive oxygen species (ROS) was determined by fluorescent‐labeled dihydroethidium (DHE). Frozen slices (15‐μm thick) were incubated in DHE for 30 min at 37°C in a dark humidified chamber. The sections were washed with PBS three times and stained with DAPI (Beyotime Biotechnology) to stain the cell nuclei. Immunofluorescent images were taken with a fluorescence microscope (Nikon) and analyzed by using the Image J software to obtain the mean fluorescence density of each visual field.

### 4.12 Statistical analysis

Results from the experiment were expressed as means ± SD and analyzed using SPSS software. Normality of distribution was assessed by the Lilliefors test, and homogeneity of variance was tested with Levene's test. Differences between groups were determined by a one‐way ANOVA test, followed by Dunnett's *t* test or Tukey's test for post hoc comparisons. The prior level of significance was established at *p* < 0.05.

## CONFLICT OF INTEREST

No potential conflicts of interest needed to be disclosed.

## AUTHOR CONTRIBUTIONS

Ruili Dang, Mengqi Yang, Changmeng Cui, Changshui Wang, Wenyuan Zhang, Chunmei Geng, and Wenxiu Han contributed to the data acquisition; Pei Jiang conceived the study and critically revised the reports; Ruili Dang and Mengqi Yang contributed to the data acquisition; Ruili Dang, Mengqi Yang, and Pei Jiang participated in writing and revising the manuscript. All authors read and approved the final manuscript.

## Supporting information

Fig S1‐S2

Table S1

## Data Availability

The datasets used and analyzed during the current study are available from the corresponding author on reasonable request.
