# Supplementary material for: Activation of angiotensin‐converting enzyme 2/angiotensin (1–7)/mas receptor axis triggers autophagy and suppresses microglia proinflammatory polarization via forkhead box class O1 signaling
Source: Aging Cell. 2021 Sep 16;20(10):e13480. doi: 10.1111/acel.13480 (PMC8520723; doi:10.1111/acel.13480)
Supplement: Supplementary file 1 — Fig S1‐S2 [file ACEL-20-e13480-s002.docx]

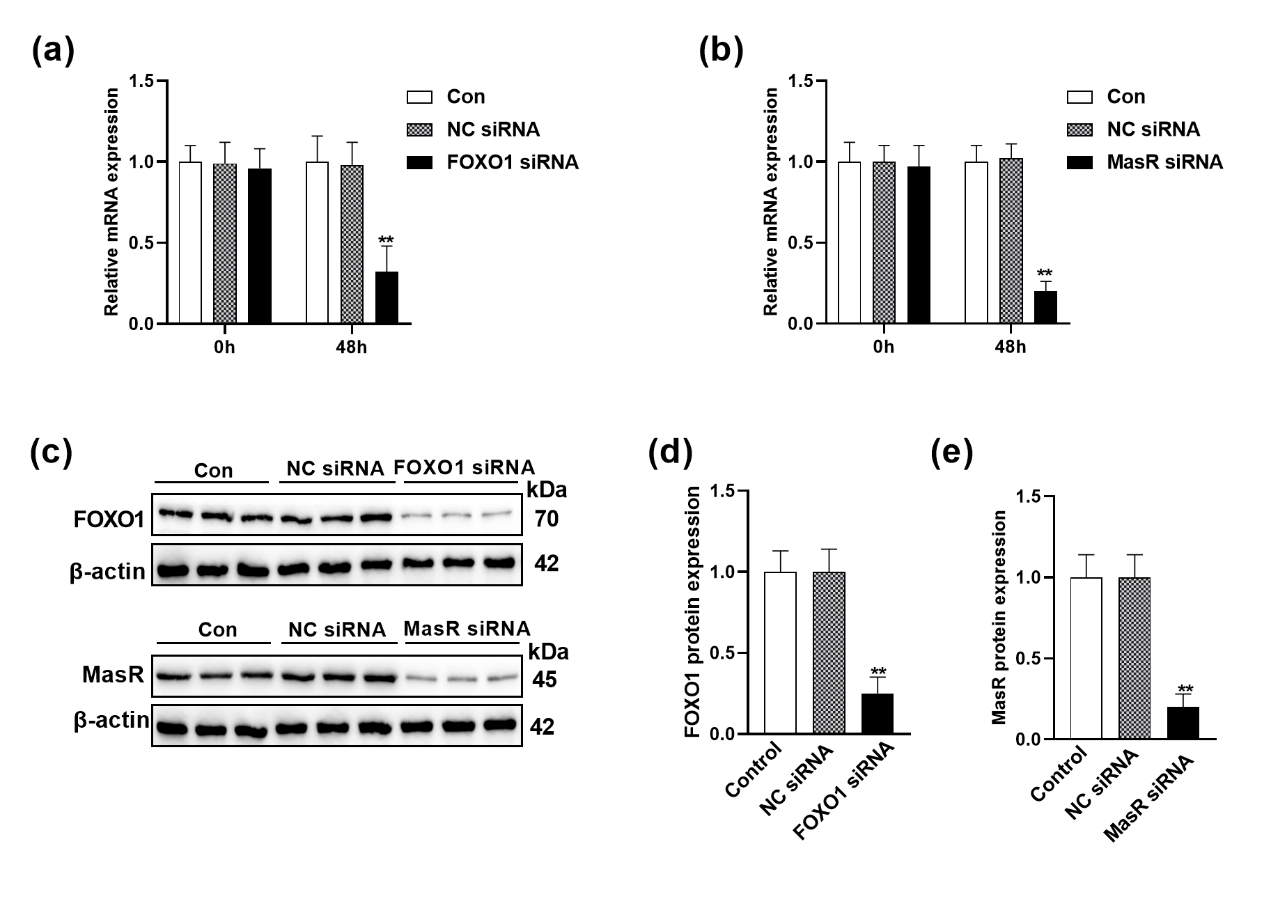


**FIGURE S1.** FOXO1 siRNA and MasR siRNA efficiently suppressed the target genes, respectively. (a, b) FOXO1 mRNA and MasR mRNA were not affected by Nc siRNA, but were markedly inhibited by FOXO1 siRNA and MasR siRNA, respectively. (c-e) Representative western blots (c) and statistical graphs of FOXO1 (d) and MasR (e) expression after 48h siRNA treatment. Data are means ± SD (n=5). **p < 0.01 compared to control group.


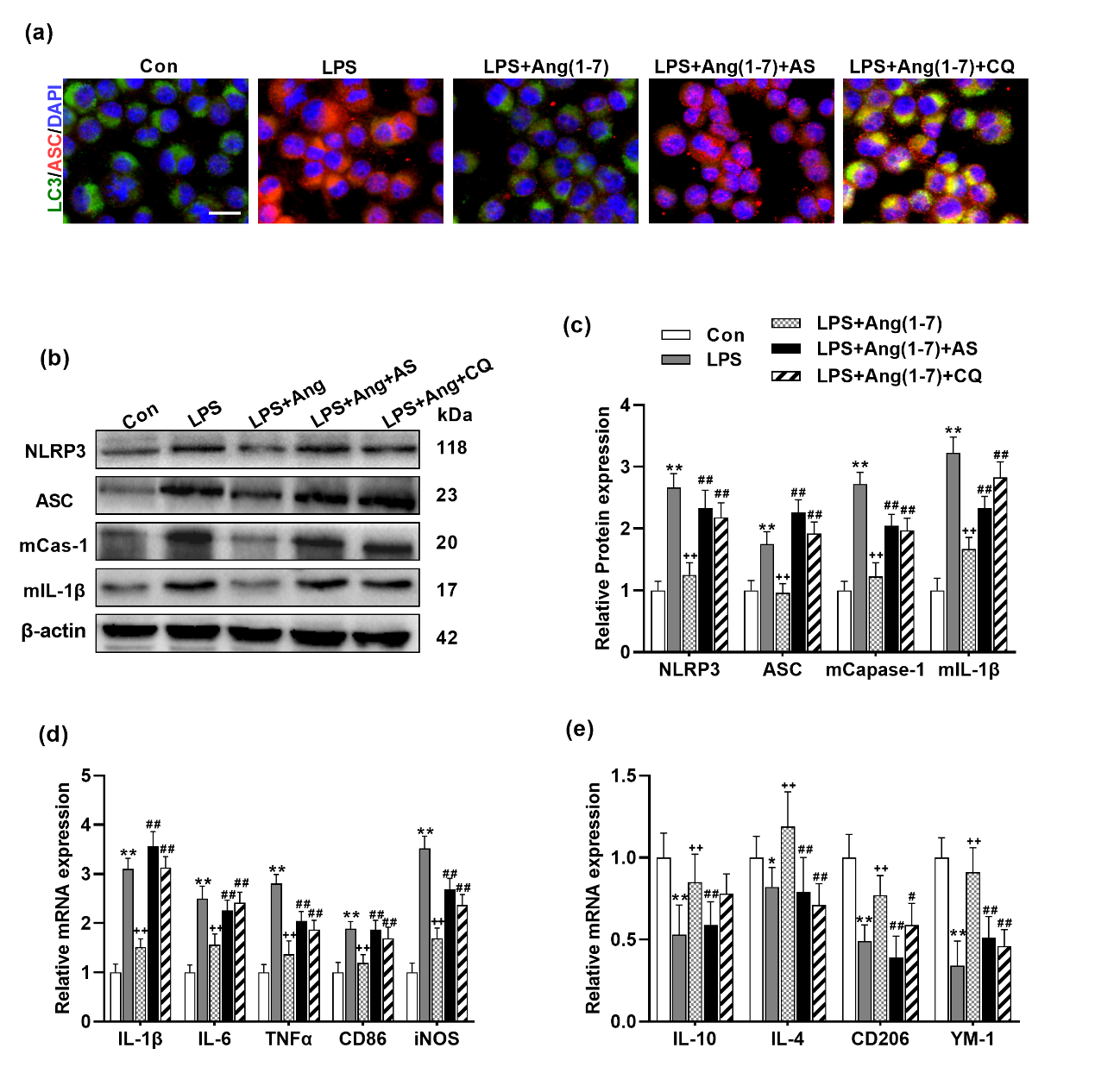


**FIGURE S2.** Inhibition of FOXO1 or autophagy induced NLRP3 accumulation and abrogated immune-regulatory effect of Ang(1-7) in BV2 microglial cells. (a) Representative images of immunofluorescence staining of LC3 (green) and ASC (red). (b, c) Representative western blots (b) and statistical graphs (c) of the major components of NLRP3 inflammasomes. (d, e) mRNA expression of biomarkers of microglial M1 phenotype (d) and M2 phenotype (e). Scale bar = 20μm. Data are means ± SD (n=6). *p < 0.05, **p < 0.01 compared to control group. ++p < 0.01 compared to LPS group. ^#^p < 0.05, ^##^p < 0.01 compared to LPS+AVE group.
