# Supplementary material for: Activation of angiotensin‐converting enzyme 2/angiotensin (1–7)/mas receptor axis triggers autophagy and suppresses microglia proinflammatory polarization via forkhead box class O1 signaling
Source: Aging Cell. 2021 Sep 16;20(10):e13480. doi: 10.1111/acel.13480 (PMC8520723; doi:10.1111/acel.13480)
Supplement: Supplementary file 2 — Table S1 [file ACEL-20-e13480-s001.docx]

**Table S1** Primer sequences used for the qPCR analysis

| **Gene** | **Sense Primer (5’-3’)** | **Antisense Primer (5’-3’)** | **Amplicon length** |
| --- | --- | --- | --- |
| IL-1β | GTGTCTTTCCCGTGGACCTTC | TCATCT CGGAGCCTGTAGTGC | 158 bp |
| IL-6 | TACCACTTCACAAGTCGGAGGC | CTGCAAGTGCATCATCGTTGTTC | 116bp |
| TNF-α | GGTGCCTATGTCTCAGCCTCTT | GCCATAGAACTGATGAGAGGGAG | 139 bp |
| CD86 | ACGTATTGGAAGGAGATTACAGCT | TCTGTCAGCGTTACTATCCCGC | 147 bp |
| iNOS | GAGACAGGGAAGTCTGAAGCAC | CCAGCAGTAGTTGCTCCTCTTC | 127 bp |
| IL-10 | CGGGAAGACAATAACTGCACCC | CGGTTAGCAGTATGTTGTCCAGC | 130 bp |
| IL-4 | ATCATCGGCATTTTGAACGAGGTC | ACCTTGGAAGCCCTACAGACGA | 125 bp |
| CD206 | GTTCACCTGGAGTGATGGTTCTC | AGGACATGCCAGGGTCACCTTT | 116 bp |
| YM-1 | TACTCACTTCCACAGGAGCAGG | CTCCAGTGTAGCCATCCTTAGG | 135 bp |
| LC3b | GTCCTGGACAAGACCAAGTTCC | CCATTCACCAGGAGGAAGAAGG | 119 bp |
| Beclin-1 | CAGCCTCTGAAACTGGACACGA | CTCTCCTGAGTTAGCCTCTTCC | 132 bp |
| ATG7 | CCTGTGAGCTTGGATCAAAGGC | GAGCAAGGAGACCAGAACAGTG | 147 bp |
| ATG12 | GAAGGCTGTAGGAGACACTCCT | GGAAGGGGCAAAGGACTGATTC | 157 bp |
| ATG5 | CTTGCATCAAGTTCAGCTCTTCC | AAGTGAGCCTCAACCGCATCCT | 107 bp |
| SOD | GGTGAACCAGTTGTGTTGTCAGG | ATGAGGTCCTGCACTGGTACAG | 114 bp |
| CAT | CGGCACATGAATGGCTATGGATC | AAGCCTTCCTGCCTCTCCAACA | 132 bp |
| β-Actin | CATTGCTGACAGGATGCAGAAGG | TGCTGGAAGGTGGACAGTGAGG | 138 bp |
